# Supplementary material for: The Multi-Omics Landscape and Clinical Relevance of the Immunological Signature of Phagocytosis Regulators: Implications for Risk Classification and Frontline Therapies in Skin Cutaneous Melanoma
Source: Cancers (Basel). 2022 Jul 22;14(15):3582. doi: 10.3390/cancers14153582 (PMC9331497; doi:10.3390/cancers14153582)
Supplement: Supplementary file 1 [file cancers-14-03582-s001.zip › cancers-1741792-supplementary.pdf]

cancers

MDPI

Supplementary Material: The Multi-Omics Landscape and Clinical Relevance of the Immunological Signature of Phagocytosis Regulators: Implications for Risk Classification and Frontline Therapies in Skin Cutaneous Melanoma

Jiahua Xing, Lingli Guo, Ziqi Jia, Yan Li and Yan Han *

	pvalue	Hazard ratio
ACTR3	<0.001	0.602(0.482−0.753)
AXL	<0.001	0.586(0.468−0.733)
BIN2	<0.001	0.588(0.470−0.735)
CD38	<0.001	0.540(0.432−0.676)
CIITA	<0.001	0.576(0.460−0.721)
DOCK2	<0.001	0.564(0.451−0.706)
FCGR1B	<0.001	0.596(0.476−0.746)
FCGR2A	<0.001	0.524(0.418−0.657)
FCRL3	<0.001	0.520(0.415−0.651)
GCNT1	<0.001	0.495(0.395−0.621)
GFI1	<0.001	0.612(0.489−0.765)
ICAM1	<0.001	0.593(0.474−0.742)
IKZF3	<0.001	0.557(0.445−0.697)
ITGAL	<0.001	0.587(0.469−0.734)
ITGB2	<0.001	0.596(0.476−0.745)
LCE1A	<0.001	1.744(1.363−2.232)
LCK	<0.001	0.582(0.464−0.729)
MS4A1	<0.001	0.606(0.485−0.758)
NCKAP1L	<0.001	0.585(0.468−0.732)
PIK3AP1	<0.001	0.606(0.485−0.758)
POU2AF1	<0.001	0.567(0.453−0.710)
POU2F2	<0.001	0.592(0.473−0.740)
PTPN6	<0.001	0.551(0.440−0.690)
PTPRC	<0.001	0.538(0.430−0.673)
RHOH	<0.001	0.536(0.428−0.671)
SASH3	<0.001	0.600(0.480−0.750)
SLA	<0.001	0.581(0.465−0.727)
SLA2	<0.001	0.573(0.457−0.719)
SPI1	<0.001	0.577(0.461−0.723)
SPN	<0.001	0.578(0.461−0.723)
VCAM1	<0.001	0.593(0.474−0.741)
ZNF683	<0.001	0.620(0.496−0.775)
BCL6	0.001	0.632(0.506−0.789)
BTK	0.001	0.646(0.517−0.807)
C1GALT1C1	0.001	0.631(0.505−0.788)
CS	0.001	1.563(1.247−1.958)
DOCK11	0.001	0.645(0.516−0.806)
PNMA5	0.001	0.644(0.515−0.806)
RAC2	0.001	0.628(0.502−0.786)
cell	0.001	0.629(0.503−0.786)
CD47	0.002	0.663(0.530−0.829)
FCGR2B	0.002	0.655(0.524−0.818)
MTO1	0.002	0.662(0.530−0.828)
FMNL1	0.003	0.666(0.532−0.833)
GNE	0.003	0.671(0.537−0.837)
PDCD10	0.003	0.666(0.533−0.832)
CMC1	0.004	0.671(0.535−0.842)
SPPL3	0.004	1.479(1.184−1.848)
MOB3A	0.005	0.684(0.547−0.856)
MS4A7	0.006	0.690(0.553−0.861)
PIK3R5	0.006	0.688(0.551−0.860)
IRX5	0.008	1.431(1.146−1.788)
UBE2J1	0.008	0.700(0.561−0.874)
FAR1	0.009	0.702(0.562−0.877)
HMGB2	0.010	0.704(0.564−0.880)
ISL2	0.010	0.704(0.562−0.881)
PRDM1	0.011	0.709(0.568−0.885)
ELOVL6	0.012	0.709(0.567−0.887)
NDUFAF7	0.012	0.710(0.566−0.889)
CD1C	0.014	0.719(0.576−0.898)
CLIC4	0.017	0.722(0.577−0.904)
SYT1	0.018	0.724(0.579−0.906)
TSC2	0.018	1.378(1.104−1.721)
ALAD	0.019	0.726(0.580−0.909)
SIX4	0.020	0.729(0.584−0.911)
CLDN18	0.024	0.737(0.589−0.921)
CADM1	0.026	0.740(0.593−0.925)
GNAI2	0.026	0.739(0.591−0.923)
SYK	0.030	0.747(0.598−0.932)
MECR	0.031	1.340(1.073−1.674)
CIT	0.032	1.338(1.070−1.673)
FOXO1	0.033	0.748(0.597−0.936)
SUPT20H	0.036	0.753(0.603−0.940)
USP22	0.036	1.328(1.063−1.659)
NUP153	0.037	1.329(1.062−1.664)
SPIB	0.037	0.753(0.602−0.942)
OSTC	0.039	0.756(0.605−0.945)
FDX1	0.041	0.759(0.608−0.948)
SIRPA	0.041	1.319(1.055−1.648)
AIP	0.043	0.759(0.607−0.949)
NDUFAF5	0.043	0.756(0.603−0.949)
SAMD4B	0.045	1.312(1.050−1.640)
CDK2	0.047	1.310(1.048−1.639)
NDUFV1	0.047	1.309(1.047−1.635)

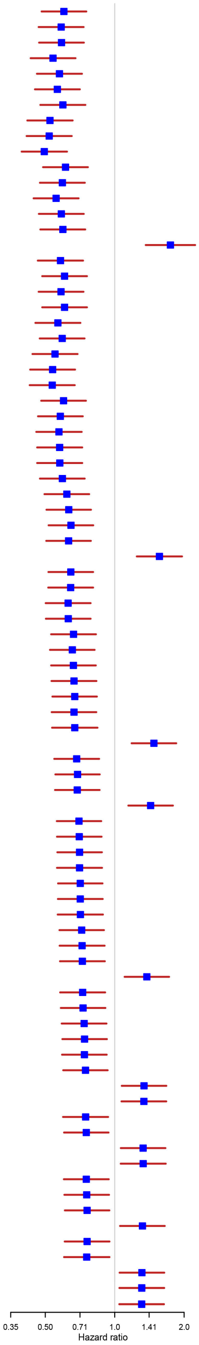

Figure S1. Forest plot of 84 genes significantly associated with prognosis. We screened 84 genes significantly associated with survival using univariate Cox regression, with 13 genes having HR > 1 and 71 genes having HR < 1.

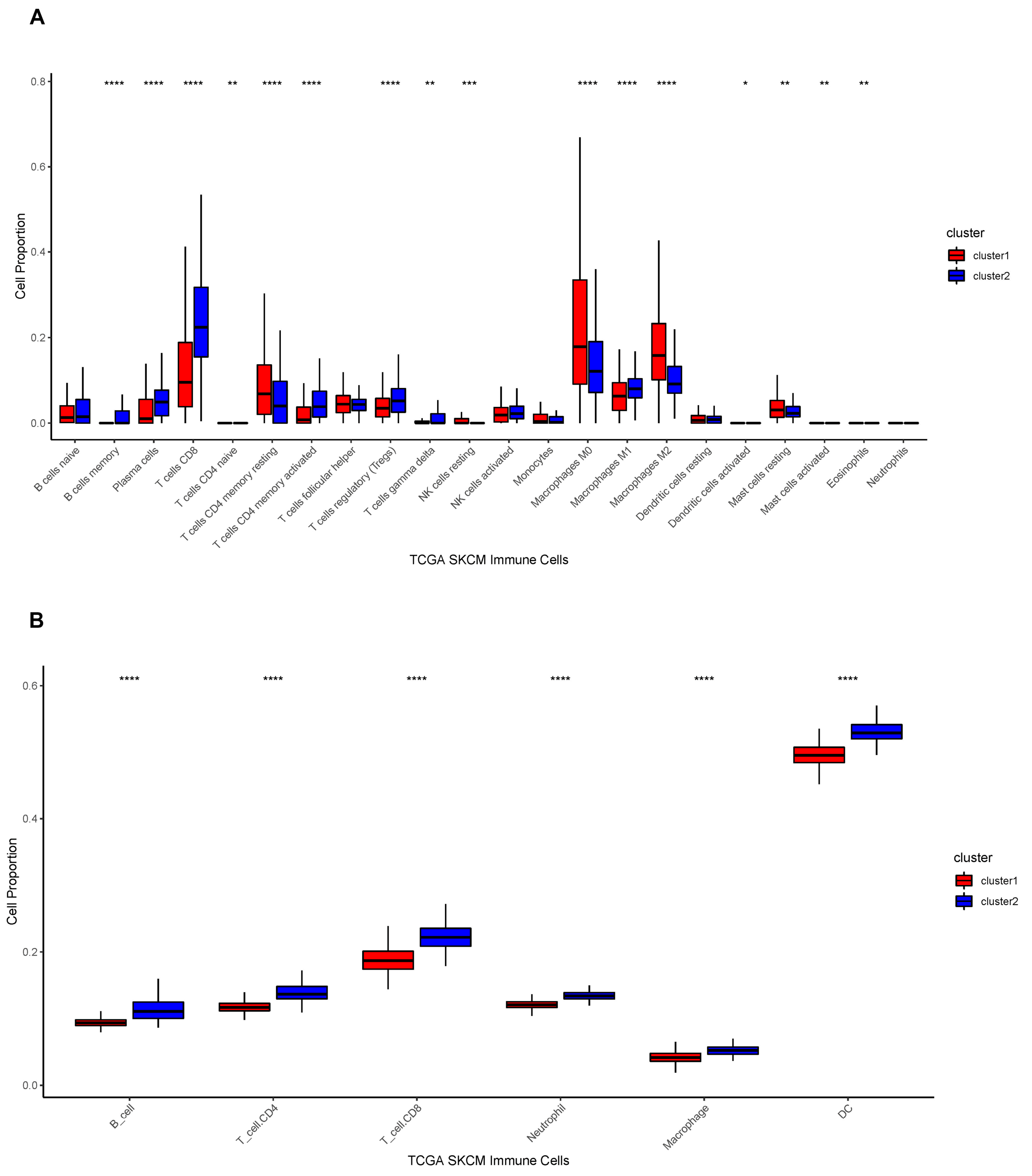

Figure S2. Calculating the proportion of immune cells in cancer samples by different methods. (A) Calculating the proportion of immune cells in cancer samples using the R package CIBERSORT. (**B**) Calculating the proportion of immune cells in cancer samples using the online database TIMER.

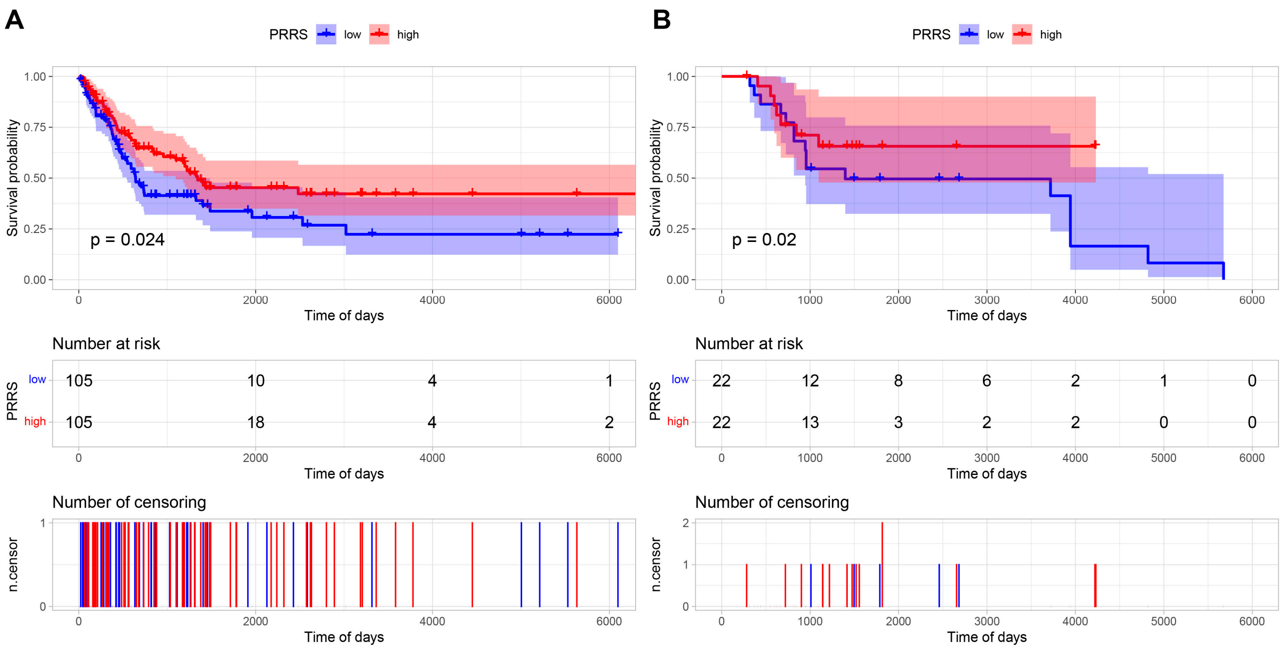

Figure S3. Validation of PRRS in other datasets. (A) The performance of PRRS in the dataset GSE65904 was validated, and the results showed statistically significant results (*P*=0.024). (B) The performance of PRRS in the dataset GSE19234 was validated, and the results showed statistically significant results (*P*=0.02).

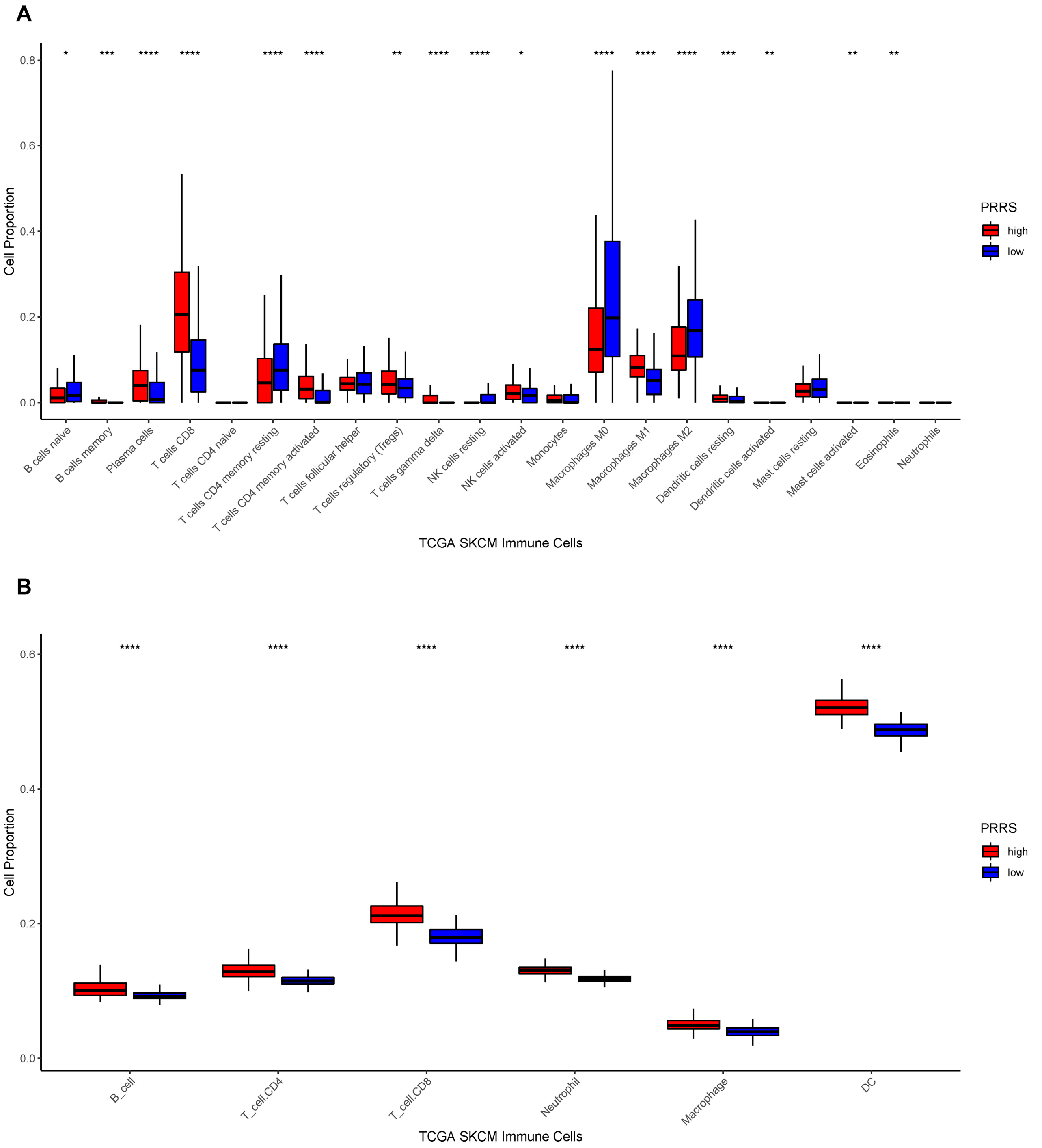

Figure S4: Analysis of differences in the proportion of immune infiltrating cells between high and low PRRS groups by different methods. **(A)** Analysis of differences in the proportion of immune infiltrating cells between high and low PRRS subgroups using CIBERSORT**.** (**B**) Analysis of differences in the proportion of immune infiltrating cells between high and low PRRS subgroups using the online database TIMER.

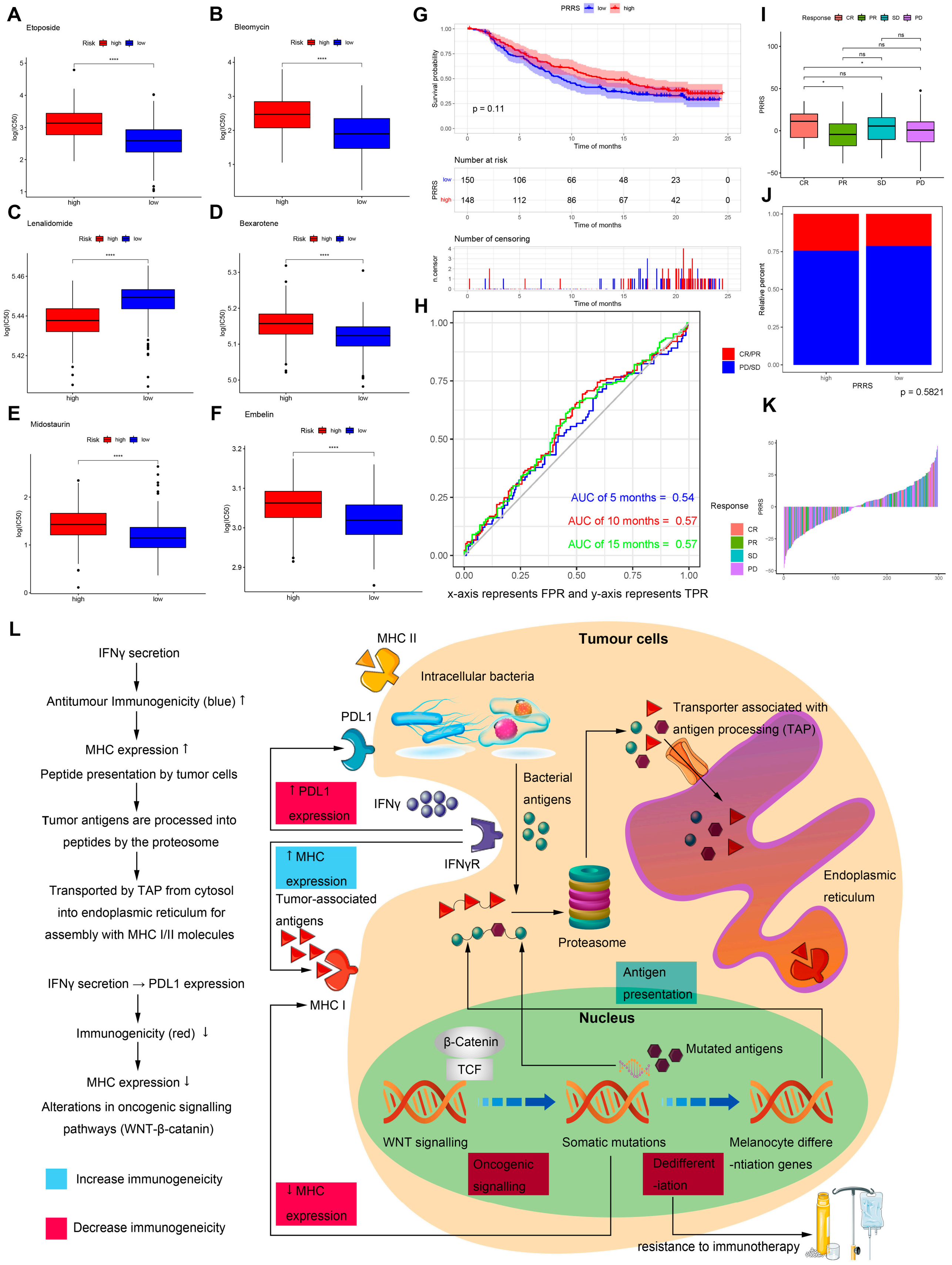

Figure S5: Potential treatment strategies based on PRRS. (**A–F**) Prediction of drug resistance to difFigure 138. drugs, taking six significantly different drugs and typical for presentation, including Etoposide, Bleomycin, Lenalidomide, Bexarotene, Midostaurin and Embelin (ns, not significant, **P*<0.05, ***P*<0.01, ****P*<0.001, *****P*<0.0001). (**G–K**) Risk model validation of IMvigor210 data. (**G**) Kaplan-Meier curves for high and low PRRS groups. (**H**) AUC time-dependent receiver operating characteristic (ROC) curves. (**I**) Statistics on the distribution of PRRS scores in each immune effect

classification. Our results showed that the PRRS scores of CR samples were significantly higher than those of PR and PD samples. (**J**) Distribution statistics of immunotherapy response in high and low PRRS groups. (**K**) Bar chart display of PRRS subline score for different response type samples. (**L**) Skin cutaneous melanoma intrinsic mechanisms and their effect on the antitumor immune response.

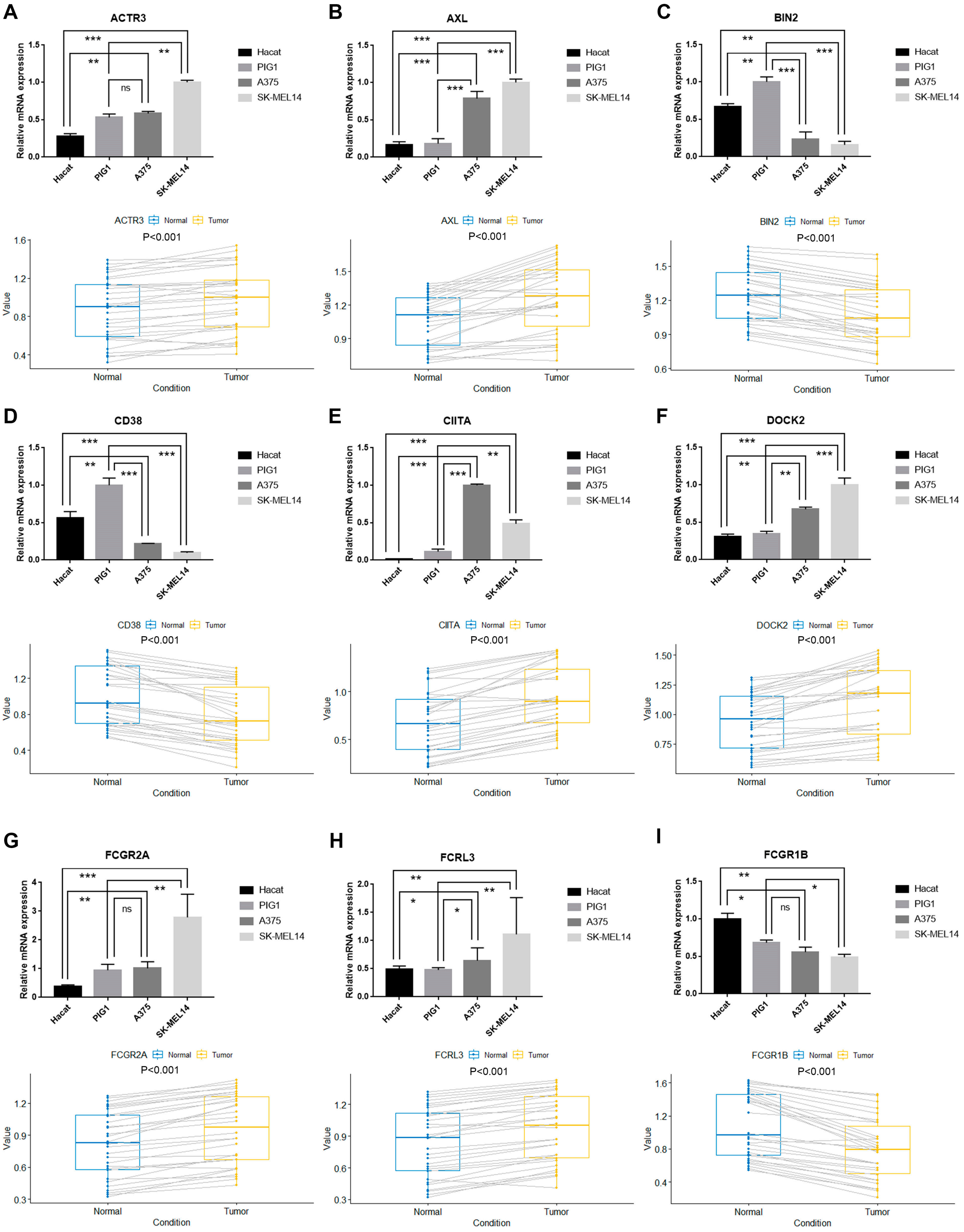

Figure S6: Validation of the mRNA expression of expression-based regulators of survival significantly associated with phagocytosis. The display of this picture contains the qRT-PCR results from four cell lines (Hacat, PIG1, A375 and SK-MEL 14), as well as the qRT-PCR results of tissue specimens from 30 patients, which taken from normal skin and SKCM. Our results included nine expression-based regulators of survival significantly associated with phagocytosis, including ACTR3, AXL, BIN2, CD38, CIITA, DOCK2, FCGR2A, FCRL3 and FCGR1B (A-I). Our results showed that the expression of ACTR3, AXL, CIITA, DOCK2, FCGR2A, and FCRL3 was significantly higher in SKCM than in normal skin tissues. The expression of BIN2, CD38, and FCGR1B was significantly lower in SKCM than in normal skin tissues (ns, not significant, **P*<0.05, ***P*<0.01, ****P*<0.001, *****P*<0.0001).

Table S1. Differences in expression of phagocytosis regulators among different subgroups (shown as significant results).

name	P age[1]	P gender[2]	P pm[3]	P pn[4]	P pt[5]	P stage[6]	P breslow[7]	P clark[8]
ABI1	0.826179	0.427429	0.297338	0.724546	0.270321	0.345915	0.221831	0.405117
ACTB	0.177925	0.903116	0.898021	0.353593	0.148032	0.948327	0.72902	0.938258
ACTR2	0.740369	0.645357	0.748661	0.914086	0.000385	0.002674	0.000213	0.035321
ACTR3	0.843407	0.94783	0.891352	0.769605	0.001345	0.000233	0.003275	0.021096
ADAM10	0.483863	0.554462	0.118791	0.669105	0.771445	0.208582	0.314034	0.288873
AIFM1	0.885945	0.545194	0.470445	0.863625	0.618138	0.320621	0.034992	0.723164
AIP	0.994604	0.266615	0.156128	0.087063	0.799077	0.489598	0.293342	0.608782
ALAD	0.873592	0.440745	0.978474	0.18848	0.300869	0.456552	0.943594	0.279263
ALCAM	0.292912	0.53504	0.801609	0.086682	0.010867	0.059606	0.002173	0.821731
AMBRA1	0.248393	0.305541	0.42999	0.114168	0.842659	0.176599	0.548444	0.975022
ANAPC7	0.689375	0.367552	0.636801	0.064876	0.058397	0.127247	0.163128	0.272784
APMAP	0.693575	0.829529	0.532662	0.966432	0.13651	0.820429	0.969481	0.98674
ARHGDIG	0.279088	0.561928	0.885845	0.13714	0.126403	0.102046	0.036106	0.236186
ARID1A	0.585809	0.561341	0.334751	0.023984	0.003416	0.000561	0.000135	0.003867
ARID1B	0.443072	0.18538	0.818603	0.041991	0.011906	0.029582	0.003091	0.117779
ARPC2	0.499104	0.28563	0.221481	0.826391	0.32989	0.127874	0.263161	0.765212
ARPC3	0.789239	0.20269	0.477735	0.287077	0.005343	0.132229	0.077977	0.114383
ARPC4	0.424674	0.148252	0.215799	0.359047	0.020826	0.646602	0.032026	0.535902
ARPC4-TTLL3	0.351251	0.631775	0.285006	0.30565	0.687605	0.845113	0.114793	0.140725
ARSB	0.40432	0.408522	0.358948	0.044479	0.129893	0.590503	0.860629	0.527274
AXL	0.790335	0.87078	0.683204	0.565536	4.31×10^{-5}	3.45×10^{-5}	7.58×10^{-7}	0.019673
BASP1	0.119093	0.483459	0.783413	0.489596	0.107959	0.003129	0.009435	0.274606
BCL6	0.055192	0.321332	0.267893	0.374179	0.1621	0.293637	0.063477	0.098955
BCL9L	0.309213	0.748526	0.302052	0.159121	0.259991	0.302401	0.531807	0.801566
BCOR	0.306175	0.592288	0.259251	0.209548	0.255557	0.103631	0.050288	0.129395
BIN2	0.068567	0.261301	0.863432	0.59512	2.48×10^{-5}	2.17×10^{-6}	3.64×10^{-5}	0.000261
BRD2	0.811793	0.958894	0.860781	0.45353	0.102588	0.321591	0.172826	0.249501
BRK1	0.120108	0.877693	0.510748	0.08261	0.127741	0.083069	0.064228	0.275784
BTK	0.007494	0.321689	0.701868	0.775787	4.73×10^{-5}	1.12×10^{-5}	0.000175	0.004193
C1GALT1	0.20196	0.559863	0.13427	0.988472	0.073474	0.056559	0.022774	0.130474
C1GALT1C1	0.87247	0.288609	0.465276	0.539899	0.459701	0.056122	0.058004	0.079906
C5AR1	0.047384	0.336175	0.190098	0.324245	0.00108	3.26×10^{-6}	5.22×10^{-5}	0.396859
CAB39	0.058716	0.634378	0.903361	0.102119	0.000298	0.000165	0.000656	0.123751
CADM1	0.714713	0.394572	0.703119	0.110067	0.144508	0.836454	0.05449	0.045957
CAPN6	0.005635	0.316163	0.907238	0.327249	0.160993	0.171635	0.074795	0.584517
CBFA2T3	0.261463	0.096193	0.386987	0.680434	0.022222	0.028231	0.213229	0.041393
CD1C	0.018873	0.124892	0.352361	0.997646	0.144433	0.024957	0.092601	0.011279
CD38	0.016173	0.060108	0.97444	0.602582	5.21×10^{-6}	3.80×10^{-8}	4.73×10^{-5}	8.65×10^{-5}
CD44	0.584341	0.365224	0.955621	0.038954	0.399799	0.003468	0.084751	0.470915
CD47	0.994604	0.587244	0.918069	0.371743	0.006349	0.031386	0.017865	0.024077

CD79B	0.015861	0.226156	0.920746	0.050361	8.15×10^{-5}	2.15×10^{-5}	0.026663	0.186995
CDK2	0.043176	0.382108	0.97982	0.578175	0.090998	0.448044	0.004676	0.596964
CEBPE	0.003429	0.467747	0.553824	0.410026	0.005281	0.000612	0.000707	0.261318
CHMP1A	0.329269	0.126246	0.286528	0.419758	0.680166	0.187568	0.347905	0.745715
CIITA	0.744674	0.503762	0.603486	0.674145	0.037386	0.008334	0.055285	0.010518
CIT	0.008979	0.018403	0.291128	0.873422	0.846037	0.508398	0.97366	0.415089
CLDN18	0.04822	0.800015	0.481395	0.064796	0.669398	0.114749	0.043666	0.701803
CLIC4	0.374141	0.558879	0.53599	0.04985	0.141376	0.18826	0.177159	0.34999
CMAS	0.439693	0.899064	0.967717	0.031299	0.766067	0.956777	0.587514	0.742313
CMC1	0.953736	0.684117	0.990582	0.359315	0.389528	0.658461	0.231086	0.854344
COX18	0.404722	0.797195	0.822537	0.933867	0.06429	0.693646	0.056089	0.760806
COX5B	0.538765	0.232958	0.813364	0.313172	0.028267	0.355361	0.347905	0.16791
CS	0.337809	0.603454	0.956965	0.754785	0.941605	0.489031	0.261377	0.322866
CXCL6	0.061348	0.119063	0.607216	0.893903	0.002603	0.13005	0.021251	0.082966
CYFIP1	0.096837	0.421915	0.943536	0.000162	0.198214	0.113094	0.053316	0.098904
DOCK11	0.040387	0.397828	0.761477	0.626715	2.28×10^{-8}	3.63×10^{-7}	2.65×10^{-8}	0.000715
DOCK2	0.146355	0.251499	0.871393	0.831011	5.79×10^{-6}	4.24×10^{-6}	3.78×10^{-6}	0.000576
DOLPP1	0.279723	0.937938	0.716931	0.529579	0.797066	0.980713	0.693878	0.149268
ELOVL1	0.002022	0.962972	0.557306	0.730551	0.000889	0.143816	4.15×10^{-5}	0.288908
ELOVL6	0.458457	0.087872	0.848873	0.246568	0.043611	0.490182	0.006134	0.310876
EMC1	0.420146	0.193267	0.966372	0.314702	0.114711	0.970473	0.539401	0.66021
EZR	0.240033	0.636985	0.46219	0.427261	0.045329	0.196827	0.061413	0.032805
FADD	0.012633	0.370279	0.539328	0.115529	0.356534	0.032212	0.004481	0.313335
FAM81A	0.143997	0.065478	0.167763	0.118161	0.240501	0.107321	0.405553	0.426816
FAR1	0.569273	0.166933	0.438912	0.355645	0.135139	0.094178	0.028357	0.259604
FCGR1B	0.80022	0.121602	0.58829	0.619312	0.018306	0.004513	0.000873	0.051266
FCGR2A	0.134667	0.052742	0.755061	0.742878	0.000109	0.000657	7.10×10^{-7}	0.062215
FCGR2B	0.584341	0.002832	0.713155	0.282908	0.060142	0.014418	0.002957	0.116631
FCRL3	0.187866	0.420859	0.727034	0.858981	7.50×10^{-6}	1.24×10^{-6}	0.000137	0.000126
FDX1	0.07732	0.464054	0.906033	0.094551	0.07509	0.319339	0.146424	0.84956
FLII	0.064146	0.020733	0.645249	0.547822	0.462872	0.663708	0.014155	0.146591
FMNL1	0.299163	0.182965	0.664731	0.530673	0.000122	3.11×10^{-5}	0.00536	0.02267
FMNL3	0.894946	0.974047	0.743554	0.718352	0.132828	0.241585	0.042161	0.183529
FOXO1	0.116419	0.767998	0.6284	0.124353	0.016919	0.054614	0.001962	0.088878
FOXO4	0.946367	0.295642	0.635598	0.023531	0.904398	0.031091	0.963632	0.608459
FUT6	0.739973	0.494	0.271583	0.453085	0.073848	0.289779	0.281467	0.662999
GAL3ST2	0.433736	0.147462	0.184609	0.956226	0.409149	0.651828	0.110648	0.915178
GAL3ST4	0.476338	0.278111	0.416323	0.151004	0.095898	0.168111	0.154757	0.872049
GCNT1	0.000385	0.878269	0.502116	0.587602	6.69×10^{-6}	3.29×10^{-7}	4.65×10^{-7}	5.89×10^{-5}
GFI1	0.071092	0.065371	0.864758	0.923932	0.001529	0.00047	0.000153	0.015544
GFI1B	0.10559	0.035617	0.276965	0.340489	0.046738	0.653049	0.074422	0.893039
GNAI2	0.458026	0.020454	0.37507	0.67047	0.841419	0.471555	0.919431	0.470104
GNE	0.346855	0.246994	0.9852	0.038249	0.002818	0.043119	0.00089	0.062695
GRHL1	0.767403	0.65483	0.472522	0.633528	0.024876	0.224099	0.004831	0.287417
GRSF1	0.458889	0.049691	0.457072	0.858746	0.297298	0.289266	0.189228	0.894568
GTPBP3	0.017852	0.470774	0.448949	0.316963	0.740352	0.278985	0.276351	0.634167
GTPBP6	0.866866	0.15073	0.645249	0.301077	0.231711	0.459112	0.809083	0.901393
GYPA	0.191836	0.755853	0.544889	0.208987	0.172733	0.443011	0.059326	0.21013
HDAC9	0.157337	0.019519	0.34327	0.968624	0.011461	0.022605	0.00148	0.023858
HDLBP	0.824516	0.600399	0.184458	0.507074	0.115779	0.347142	0.789649	0.888191
HES7	0.023989	0.164405	0.479231	0.336129	0.492014	0.035386	0.964045	0.265176
HIC1	0.060352	0.629696	0.102245	0.780725	2.58×10^{-5}	0.000427	1.15×10^{-6}	0.158774
HIGD2A	0.359029	0.005725	0.693135	0.031623	0.054521	0.432399	0.025606	0.052539

HMBS	0.304831	0.228407	0.904697	0.37945	0.71135	0.160288	0.411497	0.078369
HMGB1	0.727502	0.977545	0.954278	0.074185	0.020371	0.034626	0.01339	0.331947
HMGB2	0.187032	0.426153	0.356305	0.207682	0.00163	0.00232	0.000202	0.000487
HRC	0.004781	0.425728	0.018735	0.608995	0.123311	0.002531	0.019387	0.352759
ICAM1	0.405123	0.093019	0.316494	0.810792	0.191097	0.246376	0.099508	0.163026
IER5L	0.697784	0.257595	0.659839	0.68724	0.763969	0.755626	0.226626	0.835158
IKZF3	0.336376	0.427429	0.670867	0.712866	4.63×10^{-5}	4.50×10^{-6}	0.00024	2.80×10^{-5}
IQGAP2	0.003602	0.231529	0.728301	0.284243	0.001614	0.071961	0.000135	0.004373
IRX5	0.007321	0.345813	0.773068	0.033324	0.001093	8.86×10^{-7}	0.000106	0.440437
ISL2	0.849883	0.410756	0.781845	0.346906	0.024558	0.501253	0.000106	0.060281
ITGAL	0.105733	0.100206	0.728301	0.475498	0.000258	7.82×10^{-6}	0.00127	0.001217
ITGB2	0.076844	0.050289	0.906033	0.321303	0.000188	2.57×10^{-6}	0.00024	0.002717
JAK1	0.191108	0.462717	0.296556	0.858482	0.036744	0.023208	0.008268	0.175138
JMJD1C	0.880888	0.192268	0.797701	0.468243	2.11×10^{-5}	0.012237	1.26×10^{-5}	0.129474
KIF23	0.17701	0.014539	0.53488	0.247034	0.401634	0.062127	0.159678	0.0265
KLF6	0.011631	0.168962	0.752499	0.812956	0.89216	0.643691	0.745622	0.320898
LAMTOR2	0.144192	0.116241	0.474603	0.84202	0.249804	0.169452	0.069049	0.092615
LAMTOR4	0.024918	0.18105	0.880698	0.629224	0.000775	0.025003	0.000153	0.036854
LCA5L	0.215884	0.344319	0.170376	0.62924	0.090975	0.69776	0.038288	0.258484
LCE1A	0.264211	0.086751	0.794603	0.782277	0.000513	0.000242	1.06×10^{-5}	0.536714
LCK	0.241174	0.11189	0.97444	0.477061	0.000506	3.99×10^{-5}	0.002012	0.001965
LCMT1	0.154431	0.469875	0.205958	0.187235	0.097978	0.000659	0.050534	0.345707
LDB1	0.01404	0.931543	0.077746	0.025461	0.558128	0.001114	0.404371	0.92826
LIMK2	0.253979	0.076915	0.375979	0.243447	0.973668	0.329537	0.452632	0.500402
LIPT2	0.123367	0.373411	0.636801	0.031235	0.677777	0.264152	0.975332	0.6552
LPIN2	0.390426	0.585231	0.573272	0.76507	0.205782	0.041081	0.011412	0.087487
LRRC15	0.041797	0.354674	0.913386	0.350132	0.005078	0.175887	0.020789	0.007183
MAK	0.460617	0.53938	0.273763	0.274553	0.371854	0.359409	0.014404	0.587339
MAML1	0.692524	0.800584	0.958308	0.201312	0.07043	0.049746	0.005922	0.124207
MAML2	0.270023	0.256674	0.656179	0.162021	0.009741	0.253002	0.000339	0.086173
MAML3	0.65922	0.019788	0.58365	0.000161	0.000246	0.041046	0.001137	0.352977
MAP3K10	5.71×10^{-5}	0.399871	0.997309	0.811962	0.366158	0.056844	0.043125	0.366355
MAP3K3	0.158174	0.017551	0.432952	0.042703	0.792416	0.33012	0.556155	0.142319
MAPK1	0.042956	0.430843	0.978474	0.218566	0.462229	0.485009	0.334087	0.77535
MECR	0.036396	0.305541	0.912717	0.75887	0.022861	0.001037	0.012293	0.18736
MED13	0.974729	0.056231	0.53599	0.080425	0.000854	0.008297	0.000262	0.218707
MEX3B	0.602068	0.227561	0.395371	0.12185	0.392849	0.283789	0.036844	0.105556
MGAT1	0.931077	0.089378	0.263548	0.549823	0.036737	0.147368	0.41329	0.528599
MIB2	0.223099	0.261301	0.787303	0.585766	0.498656	0.950778	0.792071	0.534305
MOB3A	0.189662	0.05981	0.573272	0.323802	0.175476	0.021589	0.207127	0.123401
MS4A1	0.128827	0.447705	0.630791	0.399428	0.000113	1.59×10^{-5}	0.00264	0.006695
MS4A14	0.630115	0.394572	0.113698	0.90122	0.669628	0.158115	0.079763	0.353253
MS4A7	0.073807	0.277463	0.056053	0.955189	0.001285	0.00012	3.67×10^{-5}	0.046488
MSN	0.99858	0.622961	0.241871	0.284036	0.82772	0.159605	0.675395	0.564199
MTIF3	0.453724	0.441613	0.934145	0.133042	0.063598	0.347967	0.269473	0.513925
MTO1	0.984382	0.619863	0.498899	0.269174	4.79×10^{-5}	0.000106	3.42×10^{-6}	0.001654
MUC1	0.21247	0.072274	0.603486	0.63419	0.053991	0.204167	0.128145	0.566241
MUC12	0.287716	0.928057	0.034458	0.431446	0.883206	0.051721	0.471133	0.470989
MUC21	0.461179	0.77517	0.466616	0.639812	0.487665	0.785703	0.617937	0.189274
MYC	0.866866	0.417702	0.276731	0.603186	0.540088	0.544117	0.7243	0.915635
MYO9B	0.001154	0.781997	0.426059	0.559185	0.892867	0.213925	0.374354	0.872797
NANS	0.625066	0.807371	0.780823	0.22361	0.125831	0.946767	0.1292	0.77466

NCKAP1L	0.060255	0.273921	0.783413	0.505985	4.60×10^{-5}	3.40×10^{-7}	3.64×10^{-5}	0.00069
NDUFA1	0.752227	0.572729	0.276731	0.123779	0.199342	0.259202	0.020731	0.227413
NDUFA8	0.831172	0.243726	0.332222	0.525589	0.135763	0.27853	0.041633	0.660525
NDUFA9	0.405123	0.813039	0.272288	0.570684	0.404355	0.046512	0.279135	0.589688
NDUFAF3	0.126694	0.111725	0.796399	0.347686	0.095104	0.57551	0.038582	0.086483
NDUFAF5	0.80022	0.823834	0.07331	0.102222	0.006823	0.020771	0.025399	0.254937
NDUFAF7	0.614019	0.407279	0.539328	0.365269	0.056157	0.669355	0.007007	0.358381
NDUFB11	0.665416	0.012601	0.23582	0.832842	0.009055	0.334961	0.010027	0.822557
NDUFB4	0.214041	0.500502	0.956965	0.757978	0.389862	0.604847	0.066525	0.881651
NDUFB6	0.484753	0.054188	0.701868	0.696738	0.116909	0.194756	0.145843	0.092439
NDUFB9	0.357168	0.376165	0.23582	0.079483	0.009635	0.445914	0.158436	0.339087
NDUFC1	0.912425	0.890968	0.589452	0.940775	0.898891	0.192374	0.522903	0.220394
NDUFS2	0.280357	0.90717	0.946221	0.895209	0.87076	0.071111	0.523586	0.130757
NDUFS6	0.218803	0.799454	0.356305	0.007365	0.029773	0.050142	0.0959	0.469013
NDUFS7	0.067386	0.523078	0.932804	0.316129	0.229169	0.024818	0.01255	0.016483
NDUFS8	0.403518	0.142792	0.253598	0.136349	0.08021	0.037645	0.001351	0.077311
NDUFV1	0.069985	0.434702	0.42704	0.152971	0.026265	0.00156	0.000267	0.073269
NFIA	0.000845	0.960059	0.587128	0.150405	0.000478	0.012109	0.000339	0.20094
NHLRC2	0.170702	0.094451	0.77565	0.709633	0.001619	0.065453	0.000873	0.127308
NUBPL	0.756012	0.788179	0.91138	0.119939	0.523468	0.940688	0.246118	0.594375
NUP153	0.864067	0.545194	0.987891	0.580427	0.004393	0.005257	0.001298	0.203429
NXT1	0.286427	0.655358	0.663507	0.00714	0.109821	0.146113	0.249118	0.829562
OSR2	0.747368	0.01576	0.58829	0.237439	0.229934	0.609714	0.062291	0.213357
OSTC	0.000611	0.83238	0.147458	0.204244	0.018447	0.004689	0.008293	0.908969
OTUB1	0.98211	0.235257	0.045318	0.148278	0.551417	0.006718	0.277741	0.552061
OTUD5	0.475017	0.319552	0.990582	0.847874	0.112183	0.998616	0.157203	0.081254
PDCD10	0.717903	0.89328	0.903361	0.436604	0.264463	0.270326	0.108425	0.040985
PDE12	0.326102	0.854116	0.342412	0.523615	0.669219	0.418503	0.2175	0.786146
PIK3AP1	0.013737	0.287945	0.898021	0.425729	6.37×10^{-7}	3.74×10^{-8}	7.07×10^{-7}	0.000448
PIK3R5	0.119431	0.145402	0.742279	0.437478	7.46×10^{-5}	3.91×10^{-5}	7.95×10^{-5}	0.003289
PNMA5	0.079893	0.222316	0.751438	0.166734	0.023491	0.006238	0.019608	0.021782
PODXL	0.010744	0.210573	0.120797	0.514594	0.011284	0.001016	0.010943	0.249292
POU2AF1	0.131998	0.210306	0.782118	0.307541	0.000807	1.60×10^{-5}	0.017664	0.006567
POU2F2	0.019163	0.307963	0.784709	0.498382	2.62×10^{-5}	8.21×10^{-7}	1.76×10^{-5}	0.000433
PPP3CA	0.964513	0.107508	0.124475	0.109655	0.000643	0.003075	2.96×10^{-5}	0.403413
PRDM1	0.014208	0.05766	0.843591	0.415077	0.001662	3.62×10^{-5}	0.000191	0.013019
PRKCD	0.053233	0.191272	0.087275	0.743294	0.747293	0.256866	0.893683	0.979299
PTDSS1	0.168051	0.218949	0.47669	0.304042	0.447307	0.428419	0.091589	0.41163
PTEN	0.538765	0.175393	0.701868	0.156479	0.071441	0.136568	0.108194	0.227483
PTPN11	0.614019	0.08479	0.402915	0.665119	0.057351	0.098313	0.00874	0.159573
PTPN6	0.100763	0.10687	0.76791	0.578768	0.001003	6.33×10^{-5}	0.001848	0.000631
PTPRC	0.054383	0.363677	0.776943	0.871627	2.36×10^{-7}	8.46×10^{-8}	9.80×10^{-7}	5.34×10^{-5}
QPCTL	0.033806	0.700839	0.783413	0.432976	0.001982	0.10218	0.000169	0.479262
RAC1	0.473259	0.199598	0.014142	0.905773	0.605536	0.089159	0.592577	0.617155
RAC2	0.166301	0.025301	0.314868	0.200076	0.000283	1.96×10^{-6}	0.002026	0.038265
RCOR1	0.317412	0.632816	0.87405	0.229964	0.148535	0.271525	0.034631	0.605592
RHOH	0.050572	0.086521	0.966372	0.571428	2.28×10^{-5}	1.26×10^{-6}	0.000234	0.000202
RNF122	0.575569	0.619863	0.466307	0.115552	0.039057	0.124457	0.008449	0.696285
RPL21	0.510472	0.469875	0.918069	0.048894	0.0223	0.272568	0.004768	0.658488
RRAGA	0.717371	0.55055	0.973095	0.167257	0.627535	0.199528	0.893683	0.33364
RTN4IP1	0.73339	0.602435	0.700618	0.364755	0.007472	0.037595	0.018326	0.022764
SAMD4B	0.008886	0.16205	0.729568	0.845061	0.163926	0.919949	0.346829	0.682054

SASH3	0.169815	0.093162	0.940852	0.639026	0.000214	2.10×10^{-5}	0.000952	0.001306
SIRPA	0.31983	0.620379	0.735914	0.592168	0.553421	0.731038	0.093009	0.676667
SIX4	0.185136	0.093162	0.053084	0.390162	0.001625	0.010437	0.001894	0.074441
SLA	0.044366	0.356769	0.800306	0.383509	5.58×10^{-6}	1.88×10^{-6}	3.65×10^{-6}	0.000945
SLA2	0.526146	0.195024	0.710641	0.614012	0.002694	0.000293	0.004722	0.000761
SLC25A1	0.801321	0.935031	0.26211	0.711404	0.095639	0.139549	0.001496	0.179991
SLC35A1	0.033806	0.197556	0.11065	0.391599	0.044841	0.03752	0.000375	0.037801
SLC39A13	0.200454	0.221977	0.553913	0.710231	0.142113	0.017174	0.027981	0.041395
SLC39A9	0.984382	0.838088	0.485086	0.00255	0.040659	0.229389	0.549842	0.328385
SLC9A3R1	0.893258	0.096193	0.891352	0.77828	0.424778	0.194168	0.272668	0.405117
SMAGP	0.212731	0.524983	0.631995	0.908777	0.598496	0.597517	0.871348	0.938258
SMARCC1	0.356053	0.333245	0.947563	0.237441	0.041228	0.198542	0.006855	0.035321
SPI1	0.250441	0.054188	0.963684	0.229079	0.006541	0.000199	0.004706	0.021096
SPIB	0.214829	0.161174	0.425079	0.364172	0.008578	6.40×10^{-5}	0.04919	0.288873
SPN	0.257254	0.168962	0.688163	0.276664	0.00071	1.23×10^{-5}	0.001874	0.723164
SPPL3	0.233544	0.926314	0.360718	0.174405	0.144463	0.023408	0.046016	0.608782
SPTLC2	0.771756	0.815876	0.373257	0.202797	0.173445	0.202302	0.189935	0.279263
SPTSSA	0.034835	0.418122	0.172488	0.584001	0.221773	0.31581	0.192069	0.821731
SS18	0.250148	0.897328	0.097043	0.298276	0.475554	0.1808	0.035539	0.975022
ST3GAL1	0.007212	0.140613	0.265714	0.268109	5.08×10^{-5}	0.001841	7.78×10^{-5}	0.272784
ST3GAL2	0.645366	0.633336	0.690647	0.06914	0.396436	0.274343	0.045675	0.98674
ST6GALNAC1	0.380294	0.285793	0.440408	0.214064	0.302496	0.828413	0.051909	0.236186
STARD7	0.264498	0.680362	0.752499	0.454178	0.40693	0.462542	0.388033	0.003867
STK4	0.427571	0.880576	0.229223	0.942121	0.000339	2.08×10^{-5}	0.000191	0.117779
SUPT20H	0.051078	0.245505	0.895352	0.439252	2.70×10^{-5}	0.00111	0.000447	0.765212
SYK	0.058053	0.027175	0.952935	0.913526	0.000836	0.001751	0.000167	0.114383
SYT1	0.72908	0.75516	0.76274	0.158833	0.001128	0.461065	2.98×10^{-5}	0.535902
TACO1	0.017613	0.859855	0.424101	0.047159	0.022449	0.016631	0.06637	0.140725
TIMMDC1	0.075196	0.976962	0.158106	0.091262	0.10993	0.054327	0.065906	0.527274
TLE3	0.221481	0.597352	0.392564	0.339248	0.728641	0.728964	0.241454	0.019673
TM2D1	0.112498	0.359829	0.132525	0.502994	0.288413	0.58219	0.897829	0.274606
TM2D2	0.431312	0.341714	0.040636	0.409311	0.067673	0.048532	0.690012	0.098955
TM9SF3	0.173384	0.083347	0.451986	0.652455	0.010078	0.288764	0.068093	0.801566
TMEM119	0.193048	0.53938	0.711897	0.253672	0.624954	0.263019	0.775962	0.129395
TMEM165	0.641284	0.859855	0.051045	0.14323	0.084167	0.019964	0.040491	0.000261
TMEM38B	0.931643	0.37106	0.782118	0.904015	0.300291	0.192532	0.071651	0.249501
TP73	0.826733	0.103573	0.008778	0.560225	0.150511	0.091047	0.309022	0.275784
TSC2	0.375674	0.89039	0.383296	0.117337	0.569932	0.195012	0.412094	0.004193
TSPAN15	0.284821	0.343201	0.528242	0.006564	0.804519	0.554996	0.808271	0.130474
UBE2D3	0.776117	0.861004	0.812056	0.4633	0.032006	0.116396	0.004236	0.079906
UBE2J1	0.155257	0.94201	0.118393	0.895866	0.001888	3.97×10^{-5}	0.0002	0.396859
UBE2K	0.463651	0.252104	0.330543	0.719879	0.05772	0.028195	0.101462	0.123751
UBR4	0.443919	0.305886	0.460139	0.126922	0.628336	0.115258	0.547048	0.045957
UQCC1	0.238611	0.478907	0.339846	0.753582	0.653668	0.258933	0.475013	0.584517
USP22	0.879765	0.296994	0.442911	0.049824	0.604066	0.002492	0.290463	0.041393
USP34	0.360897	0.213787	0.87405	0.167136	3.08×10^{-5}	0.00443	1.17×10^{-5}	0.011279
VCAM1	0.003264	0.264731	0.859457	0.951797	7.51×10^{-7}	2.11×10^{-6}	1.10×10^{-7}	8.65×10^{-5}
VPS37A	0.78267	0.445091	0.151265	0.069109	0.151723	0.121884	0.067305	0.470915
VSIG8	0.768483	0.80963	0.870062	0.005348	0.977363	0.23527	0.990382	0.024077
WASF2	0.772846	0.011456	0.478781	0.051934	0.560452	0.180916	0.20864	0.186995
WDR1	0.058716	0.24939	0.276731	0.047683	0.430494	0.012417	0.309521	0.596964
XPR1	0.266331	0.016111	0.936827	0.228686	0.031729	0.630677	0.065139	0.261318
YBEY	0.98495	0.294293	0.266439	0.494308	0.117586	0.1976	0.077977	0.745715

ZBTB7A	0.312953	0.564301	0.878038	0.259705	0.965615	0.748773	0.568884	0.010518
ZBTB7B	0.112337	0.112716	0.684442	0.027568	0.105145	0.06472	0.006705	0.415089
ZC3HAV1	0.571207	0.823265	0.993273	0.289552	0.290947	0.653614	0.489386	0.701803
ZDBF2	0.096908	0.932706	0.864758	0.285997	0.01229	0.054132	0.000929	0.34999
ZEB2	0.672154	0.785367	0.789899	0.059641	0.344262	0.138644	0.20902	0.742313
ZFX	0.47019	4.86×10^{-8}	0.670867	0.154893	0.05839	0.031954	0.007794	0.854344
ZNF217	0.264194	0.333245	0.830418	0.763401	0.010725	0.000676	0.001874	0.760806
ZNF311	0.925986	0.070098	0.201757	0.188858	0.486605	0.46144	0.580315	0.16791
ZNF683	0.433607	0.008591	0.783413	0.215445	0.426208	0.101224	0.227634	0.322866
ZNF746	0.806277	0.866177	0.124889	0.872905	0.545733	0.287973	0.721158	0.082966

[1]Differences in the expression of phagocytosis regulators between age subgroups and the results are expressed as *P* values. If the *P*-value is less than 0.05, it means that this phagocytosis regulator has a significant difference in expression between age subgroups.

[2]Differences in the expression of phagocytosis regulators between gender subgroups and the results are expressed as *P* values. If the *P*-value is less than 0.05, it means that this phagocytosis regulator has a significant difference in expression between gender subgroups.

[3]Differences in the expression of phagocytosis regulators between pathologic_M subgroups and the results are expressed as *P* values. If the *P*-value is less than 0.05, it means that this phagocytosis regulator has a significant difference in expression between pathologic_M subgroups.

[4]Differences in the expression of phagocytosis regulators between pathologic_N subgroups and the results are expressed as *P* values. If the *P*-value is less than 0.05, it means that this phagocytosis regulator has a significant difference in expression between pathologic_N subgroups.

[5]Differences in the expression of phagocytosis regulators between pathologic_T subgroups and the results are expressed as *P* values. If the *P*-value is less than 0.05, it means that this phagocytosis regulator has a significant difference in expression between pathologic_T subgroups.

[6]Differences in the expression of phagocytosis regulators between tumor_stage subgroups and the results are expressed as *P* values. If the *P*-value is less than 0.05, it means that this phagocytosis regulator has a significant difference in expression between tumor_stage subgroups.

[7]Differences in the expression of phagocytosis regulators between breslow_depth subgroups and the results are expressed as *P* values. If the *P*-value is less than 0.05, it means that this phagocytosis regulator has a significant difference in expression between breslow_depth subgroups.

[8]Differences in the expression of phagocytosis regulators between clark_level subgroups and the results are expressed as *P* values. If the *P*-value is less than 0.05, it means that this phagocytosis regulator has a significant difference in expression between clark_level subgroups.

Table S2. The primer sequences.

Primer name	Base sequence (5' to 3')
ACTR3-F	TTCAACCATGTTCAGGGACTTTG
ACTR3-R	ACCACCACTCAATTCCTCACT
AXL-F	GTGGGCAACCCAGGGAATATC
AXL-R	GTACTGTCCCGTGTCGGAAAG
BIN2-F	GGTCGGAAACTCGTGGACTAT
BIN2-R	GAAGACATCCCTCAAGTTGGAAA
CIITA-F	CCTGGAGCTTCTTAACAGCGA
CIITA-R	TGTGTCGGGTTCTGAGTAGAG
CD38-F	CAACTCTGTCTTGGCGTCAGT
CD38-R	CCCATACACTTTGGCAGTCTACA
DOCK2-F	AGCACAAAATGTTACAGGGCA

DOCK2-R	CCATCAGATCGTACATCATGGAC
FCGR1B-F	TGGGTCAGCGTGTTCCAAG
FCGR1B-R	GTCACTTCGCCCTGAGAGAC
FCGR2A-F	TTTGAGATGAGTAATCCCAGCCA
FCGR2A-R	TCAGGCCCAGTCTCCATTTTA
FCRL3-F	AGCTCTTCCACGCCCATAGA
FCRL3-R	ATCCGAGGGTCTGGCTATCTC

Table S3. Basic information about the patients.

Number	Age	Gender	Site of lesion occurrence	TNM staging (ACJJ Staging Eighth Edition)
1	51	male	Face	T2bN0M0-IIA
2	46	male	Neck	T1aN1bM0-IIIB
3	49	male	Anterior chest	T3aN2bM0-IIIB
4	42	male	Face	T2aN0M0-IB
5	57	male	Face	T2aN1aM0-IIIA
6	52	male	Upper Extremity	T2bN1aM0-IIIB
7	42	male	Palm	T2bN0M0-IIA
8	63	male	Neck	T3bN3bM0-IIIC
9	54	male	Face	T2bN1bM0-IIIB
10	50	male	Upper Extremity	T3aN3aM0-IIIC
11	46	male	Upper Extremity	T2bN0M0-IIA
12	49	male	Face	T2bN1aM0-IIIB
13	54	male	Neck	T2bN1aM0-IIIB
14	57	male	Anterior chest	T3bN3bM0-IIIC
15	52	male	Palm	T2bN1bM0-IIIB
16	45	male	Upper Extremity	T2aN0M0-IB
17	57	male	Palm	T2bN0M0-IIA
18	59	male	Lower extremities	T3bN3bM0-IIIC
19	46	female	Palm	T2bN0M0-IIA
20	49	female	Neck	T2bN1bM0-IIIB
21	50	female	Neck	T1bN0M0-IA
22	50	female	Palm	T3aN2bM0-IIIB
23	57	female	Lower extremities	T2bN0M0-IIA
24	49	female	Face	T1aN0M0-IA
25	41	female	Lower extremities	T2aN0M0-IB
26	54	female	Face	T2bN0M0-IIA
27	46	female	Palm	T1aN0M0-IA
28	51	female	Face	T2aN1bM0-IIIB
29	50	female	Anterior chest	T2bN0M0-IIA
30	48	female	Neck	T1bN0M0-IA
